# Practising what we preach: using cognitive load theory for workshop design and evaluation

**DOI:** 10.1007/s40037-015-0221-9

**Published:** 2015-10-21

**Authors:** Laura M. Naismith, Faizal A. Haji, Matthew Sibbald, Jeffrey J. H. Cheung, Walter Tavares, Rodrigo B. Cavalcanti

**Affiliations:** 1 HoPingKong Centre for Excellence in Education and Practice, University Health Network – Toronto Western Hospital, 399 Bathurst Street, East Wing 8-427C, Toronto, ON, M5T 2S8 Canada; 2 The Wilson Centre, University Health Network and University of Toronto, Toronto, Canada; 3 Faculty of Medicine, University of Toronto, Toronto, Canada; 4 Faculty of Medicine, McMaster University, Hamilton, ON Canada; 5 Centennial College School of Health Studies, Toronto, Canada

**Keywords:** Cognitive load theory, Programme evaluation, Faculty development

## Abstract

Theory-based instructional design is a top priority in medical education. The goal of this Show and Tell article is to present our theory-driven approach to the design of instruction for clinical educators. We adopted cognitive load theory as a framework to design and evaluate a series of professional development workshops that were delivered at local, national and international academic conferences in 2014. We used two rating scales to measure participantsʼ cognitive load. Participants also provided narrative comments as to how the workshops could be improved. Cognitive load ratings from 59 participants suggested that the workshop design optimized learning by managing complexity for different levels of learners (intrinsic load), stimulating cognitive processing for long-term memory storage (germane load), and minimizing irrelevant distracters (extraneous load). Narrative comments could also be classified as representing intrinsic, extraneous, or germane load, which provided specific directions for ongoing quality improvement. These results demonstrate that a cognitive load theory approach to workshop design and evaluation is feasible and useful in the context of medical education.

## Introduction

Theory-based instructional design is a top priority in medical education. Cognitive load theory (CLT) is an example of an instructional design framework that considers learners’ working memory limits. It originated in cognitive psychology and has had particular impact in multimedia instructional design [1]. CLT is increasingly used in medical education research [2, 3], but work on translating findings to educational practice is currently limited.

We identified CLT’s complex terminology as a barrier to its widespread implementation and designed a series of professional development workshops to teach clinical educators about CLT foundational concepts. We adopted CLT as a framework for our own instructional design to both promote effective learning and model how CLT concepts could be applied in practice. We also used CLT to design an evaluation form that would allow us to move beyond judging whether our instructional design ‘worked’ to understanding why it worked and how it could be improved [4].

## Workshop design and delivery

We incorporated CLT principles into the design of two 90-minute professional development workshops for clinical educators. According to CLT [2, 3],instruction imposes two types of load on working memory: *intrinsic load*, which represents the cognitive resources required to complete the learning task, and *extraneous load*, which represents the cognitive resources applied to irrelevant distracters. If the sum of intrinsic and extraneous loads approaches or exceeds the limits of working memory, learners will have no spare capacity for *germane load*, which represents the cognitive resources used to encode new learning material into long-term memory. An optimal instructional design should thus manage intrinsic load, minimize extraneous load, and stimulate germane load [2].

To manage intrinsic load, we informally gauged participants’ prior knowledge by asking them to verbally report their previous experience with CLT. We then provided a brief presentation of foundational concepts including: (i) limitations of working memory and instructional implications; (ii) differences between intrinsic, extraneous, and germane loads and strategies for balancing each to optimize learning; and (iii) empirical evidence supporting CLT-based instructional design in medical education. The presentation was designed according to best practices described in the cognitive theory of multimedia learning [5]. We provided multiple resources to manage complexity for attendees with different levels of knowledge including a 1-page handout to reinforce essential concepts for novice participants and a list of additional resources for participants with more advanced knowledge.

To stimulate germane load, we designed a small-group activity to facilitate participants’ active application of CLT principles. Their task was to identify potential sources of cognitive load from a video example. We designed a structured worksheet to stimulate discussion about how the given learning scenario could be adapted for different learners and instructional goals. We embedded ourselves in each small group to facilitate discussion and clarify theoretical questions. We then facilitated a large group discussion, during which participants shared their insights on how CLT applied in their own settings and asked any remaining questions.

To minimize extraneous load, we provided clear instructions for each activity and used examples relevant to medical education. We also modelled expected responses during the group discussions.

## Workshop 1: Simulation

Workshop 1 focused on the application of CLT to simulation design and research. The video example showed a medical student attempting a lumbar puncture for the first time in a simulated clinical environment with multiple distractions. This workshop was delivered at the Royal College Simulation Summit in September 2014 (Workshop 1A) and at the Ontario Simulation Exposition (Workshop 1B) in December 2014.

## Workshop 2: Clinical reasoning

Workshop 2 focused on the application of CLT to workplace-based learning of clinical reasoning. The video example showed a resident attempting a case review with frequent interruptions from her clinical supervisor. This workshop was delivered at the 2nd International Montreal Conference on Clinical Reasoning in October 2014.

## Workshop evaluation

In the final 5 minutes of each workshop, all participants were invited to anonymously complete an evaluation form that we designed to measure their cognitive load and identify areas for quality improvement (Appendix 1). Two self-report scales were used to measure cognitive load. The Paas scale is a single-item measure of total cognitive load, phrased as the total amount of mental effort invested in the workshop (1 = very, very low mental effort; 9 = very, very high mental effort) [6]. The Cognitive Load Component (CLC) questionnaire contains 6 Likert-type items to separately measure intrinsic, extraneous, and germane loads. Preliminary validity evidence suggests that the CLC questionnaire is sensitive to some of the sources of cognitive load [7]. We also provided space for participants to comment on how the workshop could be improved.

For each workshop iteration (1A, 1B, and 2), we used a repeated measures analysis of variance to test for differences between levels of the 3 cognitive load components. Component type (intrinsic, extraneous, germane) was the within-subjects independent variable and the corresponding load level (from the CLC) was the dependent variable. We hypothesized that we would observe high levels of germane load, moderate levels of intrinsic load, and low levels of extraneous load. Post-hoc tests were conducted using the Bonferroni correction. For Workshop 1, we additionally used an independent samples *t*-test to assess whether extraneous load decreased between workshop iterations. For Workshop 2, we extracted and analyzed narrative responses to the question of how the workshop could be improved. Two raters independently classified each comment on the basis of whether it reflected an issue with intrinsic, extraneous, or germane load. We coded an issue as representing intrinsic load when it concerned the complexity of the course content, extraneous load when it concerned the format of instruction, and germane load when it concerned the type or amount of available practice opportunities.

## Workshop 1: Simulation

Fifteen of 18 participants (83 %) completed evaluation forms for Workshop 1A. Participants’ total cognitive load was moderately high according to the Paas scale (*M* = 6.80/9, *SD* = 1.32, Table 1). There was a significant difference in mean levels of the 3 cognitive load components on the CLC, *F*(2, 28) = 72.71, *p* < .0005, partial *η* ^*2*^ = 0.84. Post-hoc tests revealed that germane load levels were significantly higher than intrinsic and extraneous load levels, but that intrinsic and extraneous load levels were similar. We revised Workshop 1A to further reduce extraneous load. For instance, we removed a diagram from the introductory presentation that one participant commented was ‘difficult to understand’ on the evaluation form. We also decided to show the video example on a single large screen, as opposed to on laptops given to each small group, in order to reduce the extraneous load associated with low volume and distractions from other groups playing the video simultaneously.

**Table 1:** Descriptive statistics for cognitive load ratings across workshops

Measure	Workshop 1A (*n* = 15)	Workshop 1B (*n* = 13)	Workshop 2 (*n* = 31)
	Mean (SD)	Mean (SD)	Mean (SD)
Paas Total^a^	6.80 (1.32)	6.92 (1.51)	5.93 (1.41)
CLC Intrinsic^b^	4.47 (1.60)	4.38 (1.50)	4.81 (1.56)
CLC Extraneous^b^	3.47 (0.92)	2.77 (0.83)	3.42 (0.89)
CLC Germane^b^	8.40 (0.91)	8.38 (1.12)	8.06 (1.12)

*CLC* cognitive load component questionnaire.

^a^Range 1–9.

^b^Range 2–10.

Thirteen of 14 participants (93 %) completed evaluation forms for Workshop 1B. Response patterns were similar to those observed for Workshop 1A (Table 1). Cognitive load component levels differed significantly from each other, *F*(2, 24) = 81.35, *p* < .0005, partial *η* ^*2*^ = 0.87. All post-hoc tests were significant, indicating high germane load levels, moderate intrinsic load levels and low extraneous load levels. There was a significant decrease in reported extraneous load from Workshop 1A to Workshop 1B, *t*(26) = 2.10, *p* = .046.

## Workshop 2: Clinical reasoning

Thirty-one of approximately 45 participants (~ 69 %) completed evaluation forms for Workshop 2. Cognitive load component levels again differed significantly from each other, *F*(2, 60) = 109.26, *p* < .0005, partial *η* ^*2*^ = 0.79, with all post-hoc tests significant. We extracted 25 comments on how the workshop could be improved. Four comments (16 %) reflected issues with intrinsic load (e.g., ‘More on how to assess cognitive workload’); 3 comments (12 %) reflected issues with extraneous load (e.g., ‘May be more directive’); and 13 comments (52 %) reflected issues with germane load (e.g., ‘More hands-on examples/videos/exercises’). Five comments (20 %) were classified as neutral (e.g., ‘At this stage of my learning seems appropriate’).

## Discussion

We used CLT as a framework to design and evaluate professional development workshops on how to apply CLT in medical education. Results suggest that our instructional design optimized learning by managing intrinsic load, stimulating germane load, and minimizing extraneous load. Our CLT-based approach to evaluation also provided specific guidance for improving the workshop. For example, the revisions made following Workshop 1A resulted in significantly lower reported levels of extraneous load in Workshop 1B. Though germane load was high for all workshop iterations, many participants reported a desire for additional examples and practice opportunities. To address this issue, we are currently expanding our workshop into a 3-hour pre-conference offering.

This was a small-scale programme evaluation carried out in a local context so we acknowledge that our evaluation results may not generalize to other programmes. The consistency of our results across settings demonstrates, however, that a cognitive load theory approach to the design and evaluation of professional development interventions is both feasible and potentially useful in the wider medical education context. In particular, this report illustrates how a short theory-based evaluation form can help us progress beyond evaluating participant satisfaction to developing an understanding of how and why a workshop ‘worked’ and how it might be further improved [4].

### Ethical approval

This programme evaluation study was exempted from ethics review by the Research Ethics Board at the University of Toronto.

### Declaration of interest

The authors report no declarations of interest.

### Attribution of research

This programme evaluation study was conducted under the auspices of the Faculty of Medicine, University of Toronto, Canada.

### Sources of support

None.
